# Intrastromal voriconazole as successful adjunctive approach for recalcitrant deep fungal keratitis

**DOI:** 10.22336/rjo.2023.3

**Published:** 2023

**Authors:** Atul Bhirud, Avinash Mishra, Mohini Agrawal, Jyoti Sharma

**Affiliations:** * Department of Ophthalmology, Military Hospital, Jalandhar, Punjab, India; ** Department of Pathology, Military Hospital, Amritsar, Punjab, India

**Keywords:** recalcitrant, fungal keratitis, intrastromal, voriconazole, conventional antifungals

## Abstract

**Aim:** To assess efficacy of intrastromal voriconazole (ISV) in the treatment of fungal keratitis non-responding to conventional antifungals.

**Methods:** Eighteen patients with smear positive fungal keratitis, not responding to conventional topical/ systemic antifungal therapy up to 2 weeks, were included in the study. Afterwards, they were given ISV (50 µg in 0.1 ml) around the ulcer and continued to receive conventional antifungal therapy. Responses to treatment (decrease in size of the ulcer and infiltrates) were recorded daily for 3-days, at 1-week and every 2 weeks for 3-months, or until the ulcer had healed completely.

**Results:** The mean age at presentation was 51 ± 17.83 years. The most common organism isolated was *Fusarium* (17/ 18), followed by *Aspergillus* (1/ 18). All the patients were successfully treated in terms of corneal healing, but one case did not improve in vision due to the existence of diabetic macular oedema. 6 patients improved after a single injection, 7 had to receive 2 and 5 improved after 3 injections. The mean number of injections in 17 treated patients was 1.94 ± 0.78. Moreover, the mean resolution time was 18.50 ± 6.25 days. The size of ulcer and height of hypopyon at presentation were noteworthy risk-factors linked to management outcomes. Deeper ulcers required a greater number of injections when compared to superficial ulcers. The mean best-corrected visual acuity improved from 0.94 to 0.25 at 3 months follow-up in all the patients.

**Conclusion:** Intrastromal Voriconazole (50 µg/ 0.1 mL) appears to be an effective adjunct therapy in cases of recalcitrant deep fungal keratitis non-responding to conventional antifungals. Though, some may require repeated injections, timely ISV administration certainly reducing the need for tectonic/ therapeutic keratoplasty.

**Abbreviations:** ISV = Intrastromal Voriconazole, AS-OCT = anterior-segment optical coherence topography, KOH = potassium hydroxide, BCVA = best-corrected visual acuity

## Introduction

Fungal corneal infections are accountable for almost 50% of cases of culture positive infections primarily in developing countries [**1**,**2**]. The risk-factors include vegetative trauma, use of contaminated contact lens and its solution, ocular surface diseases, topical steroids or antibiotics and immunosuppressive status [**3**,**4**]. In view of its delayed presentation and non-availability of antifungal agents, it has a dreaded occurrence, leading to ocular complications like corneal descemetocele, staphyloma, endophthalmitis, melting, perforation and blindness. The dealing of fungal keratitis is challenging due to quite a few limitations of available topical antifungals like sub-optimal cornea penetration, ocular surface toxicity and limited spectrum [**5**]. Also, surgical intervention, like therapeutic keratoplasty, is frequently required for deep fungal keratitis, but the success is restricted by various factors like graft rejection, recurrence of infection and limited donor corneas. 

Newer antifungals, such as Voriconazole, Posaconazole and Caspofungin, have a good safety profile and better corneal penetration when compared to conventional antifungals, such as Natamycin, thus improving treatment outcomes.

Targeted drug delivery can attain satisfactory drug concentrations at the site of the infection and the intrastromal injection of antifungal drugs has shown promising outcomes [**6**-**8**]. 

The use of intrastromal amphotericin-B and voriconazole for deep fungal keratitis non-responsive to conventional treatment has been described in anecdotal reports [**6**].

Thus, this novel study was performed to evaluate the clinical outcomes of intrastromal voriconazole (ISV) injections as an adjunctive treatment modality in 18 patients from North-East India with recalcitrant deep fungal keratitis, showing no improvement with conventional antifungal therapy.

## Methods

The study was performed at a tertiary eye care center, North-East India, from January 2022 to October 2022, was approved by the Institutional Ethics Committee and was carried out in agreement with the “Declaration of Helsinki”. A written informed consent was taken from all the patients. Therefore, patients with smear and culture positive deep fungal keratitis, not responding to topical and systemic antifungal agents including 5% Natamycin and 1% Voriconazole over a period of 2-weeks, were included in the study. 

Patients having ulcer with adjacent scleral involvement, perforation, associated features of endophthalmitis, allergic to drug and one-eyed patients were excluded from the study.

At first presentation, each patient underwent a complete ophthalmological evaluation including medical history, Snellen visual-acuity testing and slit-lamp bio-microscopy. 

The parameters recorded were the size of ulcer, size of infiltrates, any satellite lesion, pigmented lesion and height of hypopyon if present. The area of the ulcer was calculated based on its maximum diameter. The depth of corneal involvement in all the cases was extended up to or deeper than mid-stromal level. Anterior-segment optical coherence topography (AS-OCT) was performed to confirm the depth of ulcers and the B-scan to rule out posterior pathology including endophthalmitis. The diagnosis of fungal keratitis was made based on clinical evaluation and positive smear and culture.

Corneal scraping was done under topical anaesthesia (0.5% proparacaine hydrochloride) and sent for microbiological investigation like potassium hydroxide (KOH) stain, Gram stain and cultures on blood agar, chocolate agar and Sabouraud-dextrose agar. 

Topical antifungal was started as soon as the fungus was recognized positive by smear. The initial therapy included topical 5% Natamycin 2-hourly, 2% homatropine sulphate and 0.5% moxifloxacin.

“No response” was defined as “no change in the size of the ulcer/ infiltrates”; “worsened” defined as “increase in size/ depth of ulcer/ infiltrate by 20% or impending perforation”; and “healing” defined as if the area or size of the ulcer and infiltrates reduced by ≥ 20% from initial presentation.

If no response to therapy was seen or ulcer showed signs of worsening, topical 1% Voriconazole 1-hourly and oral ketoconazole 200 mg twice daily, were added to the treatment. If the patients showed “no response” to this combined therapy in another two weeks, they were then given an injection of intrastromal Voriconazole (ISV 50 µg/ 0.1 ml) being injected around the fungal ulcer.

ISV was available as 200 mg of white lyophilized powder in a vial. It was reconstituted with 19 ml of lactated Ringer solution to attain 20 ml concentrate comprising 10 mg/ ml of Voriconazole. A 1 ml aliquot part of this solution was further diluted with 20 ml of lactated Ringer solution to a get a concentration of 0.5 mg/ ml (50 μg/ 0.1 ml). This reconstituted solution was filled in a 1 ml tuberculin syringe with a 30-gauge needle. The preloaded drug was administered under peribulbar anaesthesia and full aseptic conditions. With the bevel down, the needle was introduced obliquely from the clear area to reach the infiltrate at the mid-stromal level in each case.

The drug was then injected. Five divided doses were administered around the infiltrate in such a manner that a centripetally directed, progressive wave of fluid appeared to incorporate the infiltrate along each meridian. Circumferential ISV injection warranted the formation of a barrage of ISV around the whole infiltrate area. Intraoperative complications were recorded if any.

Post intrastromal injection, all patients were continued on previously prescribed topical antifungal therapy. Patients were examined daily for 3 days, then at 1-week followed by every 2 weeks for 3 months or until the ulcer healed completely. The response was recorded in the form of best-corrected visual acuity (BCVA), size of ulcer/ infiltrate, height of hypopyon, satellite lesions or any complication using slit-lamp. The infection was considered “resolved” when there was complete healing of the epithelial defect with resolution of infiltrates and a scar was formed. The patients were continued on topical antifungal therapy for at least one week, even after the complete resolution of the infection. In case of deteriorating or no response within 3 to 7 days, the ISV was repeated. 

Patients with perforation or progression of infiltrate size by > 20% (despite 3 ISV injections), were considered as “treatment failure” and they were planned for tectonic/ therapeutic penetrating keratoplasty.

**Statistical analysis**

Data was analyzed using SPSS for Windows (version 20.0). Qualitative data variables were expressed as frequency and percentage, while quantitative data variables expressed as mean, standard deviation and median p-value < 0.05 was considered significant.

## Results

The study included 18 patients. There were 13 males (72.22%) and the rest were females. The mean age at presentation was 51 ± 17.83 years (range: 22-78 years). The most common cause found was vegetative trauma (50%). The mean initial BCVA was 0.94 ± 0.50. All patients had anterior (anterior 1/ 3rd) to mid-stromal (anterior 2/ 3rd) involvement of corneal stroma on slit lamp examination. The mean size of corneal ulcer measured along the longest axis was 4.44 ± 1.27 mm (range: 2-7 mm). The mean depth of corneal ulcer measured with AS-OCT was 306.5 ± 51.52 μm (range: 390-188 µm). 13 patients (72.22%) had satellite lesions on cornea while 3 patients (16.67%) had hypopyon. Considering the location of corneal ulcer, 3/ 18 patients (16.77%) had centrally located ulcer, 9/ 18 (50%) had paracentral ulcer and 6/ 18 patients (33.33%) had peripherally located ulcer. The most common organism isolated was *Fusarium* found in 17 patients (94.44%), followed by *Aspergillus* seen in only 1 patient (5.55%).

All the patients (100%) were successfully treated in terms of corneal healing, but one patient did not improve in terms of vision due to diabetic macular edema. 

Thus, out of 18 patients, 17 patients (94.44%) were successfully treated with ISV. 6 patients improved after a single injection, 7 patients needed 2, while 5 patients improved after 3 injections. The mean number of injections in 17 cured patients was 1.94 ± 0.78. The mean resolution time was 18.50 ± 6.25 days (range: 12-36 days). 

**Fig. 1:**
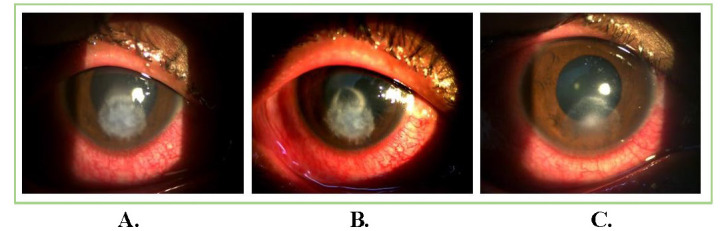
Patient with non-resolving and non-responding fungal corneal ulcer; (**A**) Stromal infiltrate before intrastromal voriconazole injection; (**B,C**) Healing of corneal ulcer of regular follow-ups after intrastromal voriconazole injection

**Fig. 2:**
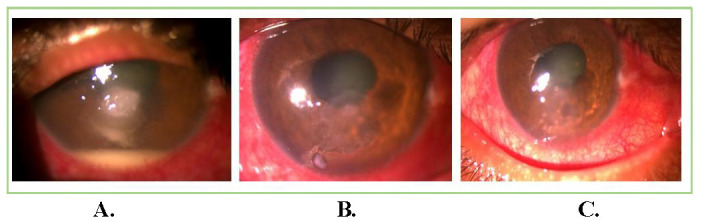
Patient with long-standing deep fungal corneal ulcer; (**A**) Non-healing recalcitrant fungal corneal ulcer with infiltrates and hypopyon before intrastromal voriconazole injection; (**B,C**) Healing of ulcer and complete resolution of hypopyon on regular follow-ups after second injection of intrastromal voriconazole

**Fig. 3:**
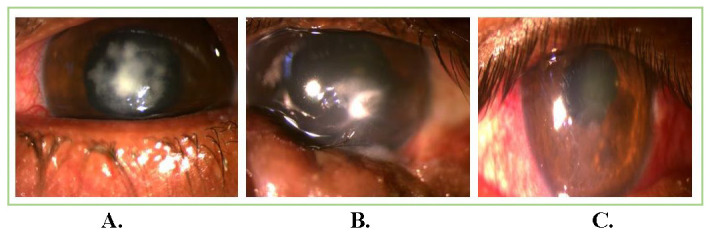
Patient with recalcitrant fungal corneal ulcer; (**A**) Non-responsive deep fungal corneal ulcer with infiltrates before intrastromal voriconazole injection; (**B,C**) Healing of ulcer and complete resolution on regular follow-ups after targeted delivery of intrastromal voriconazole

**Fig. 4:**
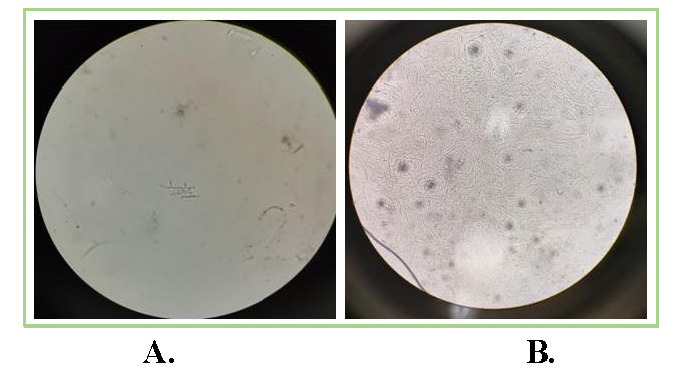
KOH mount of two patients showing filamentous fungi (**A,B**) depicting septate, branched filament at 90 degrees suggestive of Fusarium species

13 patients with satellite lesions and 3 patients with hypopyon resolved completely after injections. In our study, no case had perforation before or after the administration of ISV. Residual corneal depth after healing was 422.06 ± 88.89 μm. The mean astigmatism after healing and follow-up at 3 months was 1.49 ± 0.69 diopter. The size of the ulcer at presentation and height of hypopyon were noteworthy risk-factors associated with treatment results. The deep ulcers demanded a greater number of injections when compared to superficial ulcers. Final BCVA (excluding the patient who had diabetic macular edema) was 0.25 ± 0.43 at 3 months follow-up. **Fig. 1**-**3** show examples from our study depicting clinical resolution of deep fungal keratitis after ISV on follow-ups. No intraoperative drug related side effects were noted. **Fig. 4** shows filamentous fungi on KOH mount of two patients.

## Discussion

Fungal keratitis is a vision-threatening corneal infection that is managed by anti-fungal agents. These drugs can be used topically or orally, but have limitations. None of the currently available antifungal agents, including Voriconazole, are able to manage deep fungal keratitis effectively because of factors like sub-optimal penetration, ocular surface toxicity and their limited spectrum and clinical response [**9**]. 

Among antifungals, Voriconazole has advantages like optimum activity against fungi that are resistant to amphotericin-B, good safety profile and has shown to be effective against *Fusarium* and *Aspergillus* [**10**-**13**]. 

Up until now, intrastromal amphotericin-B has been used to treat recalcitrant fungal ulcers [**14**]. Yet, some complications, like ocular surface and retinal toxicity, have been reported with amphotericin-B. Voriconazole has shown better outcomes due to lower mean inhibitory concentration against filamentous fungi and better penetration [**5**,**15**].

As recent modalities of targeted drug delivery are needed [**6**], targeted delivery of voriconazole via intrastromal injection might help to accomplish adequate drug concentration at the site of infection in non-healing fungal keratitis [**7**-**10**]. 

In our study, the non-responding deep ulcers, even after 2 weeks of conventional topical antifungal therapy, were taken for ISV. The drug was given along the circumference of the infiltrates in the dose of 50 µg/ 0.1 ml. 

Previous studies from Western and Southern India documented *Fusarium* as the most common fungus causing keratitis [**7**,**16**]. In our study, except for one case, *Fusarium* was found in all the rest of the cases.

Our study was supported by previous literature data such as the work of Sharma et al. that reported > 80% success rate when 12 patients with recalcitrant fungal keratitis were treated with additional ISV and the mean healing time of 10 healed eyes was 39.75 ± 7.62 days [**8**]. Moreover, patients who presented early had a lesser number of injections and healed faster when compared to those presenting in later stage. Also, patients with a smaller ulcer and infiltrate, required a lesser number of injections. But, a statistical correlation could not be made because of the small sample size. Another study by Kalaiselvi et al. reported a mean resolution time of 45.68 ± 11.49 days in 17/ 25 eyes after ISV [**7**]. In our study, there was a 100% success rate in corneal healing after ISV and the mean healing time was also much lesser (18.50 ± 6.25 days) when compared to previous studies.

In contrast to our reports of ISV being a successful treatment in 18 cases of recalcitrant deep fungal keratitis, Masanori et al. reported ISV as an ineffective approach in treating filamentous fungal keratitis [**17**]. A randomized study also concluded that intrastromal injections had no valuable effect over topical therapy [**18**]. Even if the sensitivity of ISV to different fungal genus remains unknown, this adjunctive therapy may be started in selected patients who do not respond to conventional antifungal agents.

Intraocular voriconazole is used as an off-label drug in treating fungal keratitis. ISV might be used in severe fungal keratitis, in which a higher concentration of the drug is warranted at the site of infection. ISV might be a safe and cost-effective method of providing a higher concentration of the drug in cases at risk of corneal perforation. Repeated intrastromal injections of voriconazole have also been tolerated with no long-term ocular toxicity [**19**]. Our study also showed improvement in all the cases, with no adverse effects after the use of ISV. However, further studies relevant to ISV are warranted to regulate the optimal concentration of voriconazole with minimal toxicity to the cornea.

In our study, the mean time duration between the onset of symptoms and presentation to the hospital was 10.8 ± 12.46 days. This duration was similar to some of the studies performed earlier in the Indian scenario [**7**]. Requirement for re-injections for optimum resolution has been described by earlier studies [**7**,**8**]. Sharma et al. also reported that 10/ 12 eyes required ≥ 2 injections to attain optimal response. Similarly, in our study, repeated injections were given, which helped in the healing of the ulcer in most of the cases. In our case series, no unpleasant effects of repeated injections were registered. As literature is scarce regarding the pharmacokinetics of voriconazole injection into the corneal stroma, prerequisites for reinjection and the interval between injections, all need to be evaluated on clinical grounds. However, our study demonstrated that a significant number of such patients with deep recalcitrant fungal keratitis needed repeated injections for better visual outcomes. Although ISV has shown promising outcomes, the dosage and frequency of injections are yet to be determined. Large clinical trials with large sample sizes and long term follow ups are warranted.

Studies have also found Fusarium to be resistant to many of the currently available antifungals, but, in our study, all the cases with Fusarium keratitis were treated successfully resulting in good outcomes [**7**]. 

Thus, instead of keeping this option as a last resort, it may be valuable to attempt ISV in recalcitrant deep fungal keratitis cases, including Fusarium keratitis cases, before considering keratoplasty [**20**]. 

## Conclusion

Intrastromal voriconazole (50 µg/ 0.1 mL) seems to be an effective adjuvant treatment modality for recalcitrant deep fungal keratitis. Most of the ulcers show a decent response to this targeted therapy, though few patients may demand re-injections. This will result in reducing the risk of dreadful complications and the need for tectonic/ therapeutic keratoplasty. 

**Conflict of Interest statement**

The authors state no conflict of interest.

**Informed Consent and Human and Animal Rights statement**

Informed consent has been obtained from all individuals included in this study.

**Authorization for the use of human subjects**

Ethical approval: The research related to human use complies with all the relevant national regulations, institutional policies, is in accordance with the tenets of the Helsinki Declaration, and has been approved by the Ethics Committee of the Military Hospital, Jalandhar, Punjab, India.

**Acknowledgements**

None.

**Sources of Funding**

None.

**Disclosures**

None.

## References

[1] Singh A, Singh D, Kumar S, Mishra A, Verma R, Mishra V (2015). A retrospective study of fungal corneal ulcer from the western part of Uttar Pradesh. Int J Res Med Sci.

[2] Ou JI, Acharya NR (2007). Epidemiology and treatment of fungal corneal ulcers. International Ophthalmology Clinics.

[3] Taneja M, Ashar JN, Mathur A, Nalamada S, Garg P (2013). Microbial keratitis following vegetative matter injury. International Ophthalmology.

[4] Thomas PA, Kaliamurthy J (2013). Mycotic keratitis: epidemiology, diagnosis and management. Clinical Microbiology and Infection.

[5] Müller GG, Kara-José N, Castro RS (2013). Antifungals in eye infections: drugs and routes of administration. Revista Brasileira de Oftalmologia.

[6] Prakash G, Sharma N, Goel M, Titiyal JS, Vajpayee RB (2008). Evaluation of intrastromal injection of voriconazole as a therapeutic adjunctive for the management of deep recalcitrant fungal keratitis. American Journal of Ophthalmology.

[7] Kalaiselvi G, Narayana S, Krishnan T, Sengupta S (2015). Intrastromal voriconazole for deep recalcitrant fungal keratitis: a case series. British Journal of Ophthalmology.

[8] Sharma N, Agarwal P, Sinha R, Titiyal JS, Velpandian T, Vajpayee RB (2011). Evaluation of intrastromal voriconazole injection in recalcitrant deep fungal keratitis: case series. British Journal of Ophthalmology.

[9] Jurkunas UV, Langston DP, Colby K (2007). Use of voriconazole in the treatment of fungal keratitis. International Ophthalmology Clinics.

[10] Vorwerk CK, Streit F, Binder L, Tuchen S, Knop C, Behrens-Baumann W (2008). Aqueous humor concentration of voriconazole after topical administration in rabbits. Graefe’s Archive for Clinical and Experimental Ophthalmology.

[11] Thiel MA, Zinkernagel AS, Burhenne J, Kaufmann C, Haefeli WE (2007). Voriconazole concentration in human aqueous humor and plasma during topical or combined topical and systemic administration for fungal keratitis. Antimicrobial Agents and Chemotherapy.

[12] Vemulakonda GA, Hariprasad SM, Mieler WF, Prince RA, Shah GK, Van Gelder RN (2008). Aqueous and vitreous concentrations following topical administration of 1% voriconazole in humans. Archives of Ophthalmology.

[13] Saravolatz LD, Johnson LB, Kauffman CA (2003). Voriconazole: a new triazole antifungal agent. Clinical Infectious Diseases.

[14] Garcia-Valenzuela E, Song CD (2005). Intracorneal injection of amphothericin B for recurrent fungal keratitis and endophthalmitis. Archives of Ophthalmology.

[15] Clode AB, Davis JL, Salmon J, Michau TM, Gilger BC (2006). Evaluation of concentration of voriconazole in aqueous humor after topical and oral administration in horses. American Journal of Veterinary Research.

[16] Kumar A, Pandya S, Kavathia G, Antala S, Madan M, Javdekar T (2011). Microbial keratitis in Gujarat, Western India: Findings from 200 cases. Pan Afr Med J.

[17] Niki M, Eguchi H, Hayashi Y, Miyamoto T, Hotta F, Mitamura Y (2014). Ineffectiveness of intrastromal voriconazole for filamentous fungal keratitis. Clinical Ophthalmology (Auckland, NZ).

[18] Sharma N, Chacko J, Velpandian T, Titiyal JS, Sinha R, Satpathy G, Tandon R, Vajpayee RB (2013). Comparative evaluation of topical versus intrastromal voriconazole as an adjunct to natamycin in recalcitrant fungal keratitis. Ophthalmology.

[19] Bang S, Edell E, Eghrari AO, Gottsch JD (2010). Treatment with voriconazole in 3 eyes with resistant Acanthamoeba keratitis. American Journal of Ophthalmology.

[20] Srujana D, Bista N, Agrawal M (2022). Outcomes of Descemet stripping endothelial keratoplasty in cases of corneal endothelial dysfunction. Oman Journal of Ophthalmology.

